# CT-like images based on T1-weighted gradient echo MRI sequences for the assessment of fractures of the hand and wrist compared to CT

**DOI:** 10.1007/s00256-024-04683-7

**Published:** 2024-04-25

**Authors:** N. Hesse, G. C. Feuerriegel, B. Erber, P. Reidler, V. Gottfried, Y. Stohldreier, R. Schmitt, O. Dietrich, A. S. Gersing, J. E. Spiro

**Affiliations:** 1 Department of Radiology, LMU University Hospital, LMU Munich, Munich, Germany; 2 Department of Diagnostic and Interventional Radiology, Klinikum Rechts der Isar, School of Medicine, Technical University of Munich, Munich, Germany; 3 Department of Neuroradiology, LMU University Hospital, LMU Munich, Munich, Germany

**Keywords:** Wrist, Scaphoid, Fracture, Bone, CT-like MRI, T1GRE

## Abstract

**Objective:**

To evaluate the performance of a 3D T1-weighted gradient-echo (3D T1GRE) computed tomography (CT)-like magnetic resonance imaging (MRI) sequence for detecting and assessing wrist and hand fractures compared to conventional CT.

**Methods:**

Subjects with acute wrist or hand fracture in CT underwent additional 3 T MRI including a CT-like 3D T1GRE sequence and were compared to patients without fractures. Two radiologists assessed fracture morphology on both modalities according to the Arbeitsgemeinschaft Osteosynthese (AO) and graded image quality and diagnostic confidence on a 5-point Likert scale.

Besides diagnostic test evaluation, differences in image quality and diagnostic confidence between CT-like MRI and CT were calculated using the Wilcoxon test. Agreement of AO classification between modalities and readers was assessed using Cohen’s Kappa.

**Results:**

Twenty-eight patients with 43 fractures and 43 controls were included. Image quality (3D T1GRE 1.19 ± 0.37 vs. CT 1.22 ± 0.42; *p* = 0.65) and diagnostic confidence (3D T1GRE 1.28 ± 0.53 vs. CT 1.28 ± 0.55; *p* = 1.00) were rated excellent for both modalities. Regarding the AO classification, intra- (rater 1 and rater 2, κ = 0.89; 95% CI 0.80–0.97) and interrater agreement were excellent (3D T1GRE, κ = 0.82; 95% CI, 0.70–0.93; CT, κ = 0.85; 95% CI, 0.75–0.94). CT-like MRI showed excellent sensitivity, specificity and accuracy for fracture detection (reader 1: 1.00, 0.92, 0.96; reader 2: 0.98, 0.94, 0.96).

**Conclusion:**

CT-like MRI is a comparable alternative to CT for assessing hand and wrist fractures, offering the advantage of avoiding radiation exposure.

## Introduction

Wrist and hand fractures are among the most common bone injuries [1, 2]. Due to the outstanding importance of the upper extremity as a functional and expressive organ, early diagnosis and therapy of wrist and hand fractures are essential for a positive outcome in affected patients [3, 4]. If a fracture of the wrist or hand is suspected, an X-ray examination is usually the first step in radiological diagnostics. Computed tomography (CT) is performed if the clinical picture is typical but there is no evidence of a fracture on the X-ray, or if the therapeutic procedure is to be planned. Magnetic resonance imaging (MRI) is routinely used to detect occult fractures, and to diagnose non-bony concomitant injuries, especially of ligaments and cartilage [5, 6].

Since X-ray and CT examinations are always associated with radiation exposure of the patient, fracture diagnosis using MRI alone would be desirable, especially in younger patients and those who require regular follow-up [7].

Due to its low proportion of free water and its rapid T2* relaxation, direct imaging of cortical bone using MRI has long been difficult. In recent years, however, great progress has been made in the development of new MRI sequences, which have improved the representation of bony structures to such an extent that it is now possible to acquire so-called CT-like images [7, 8]. Their potential for detecting bony fractures, degeneration and osseous pathologies has already been proven in various studies, in particular, in the area of the spine, skull, mandibula, shoulder, knee and tibia [9–16].

The aim of our study was to evaluate the performance of CT-like MRI images derived from a T1-weighted gradient echo (T1GRE) sequence for the detection and classification of fractures of the wrist and hand compared to CT as standard of reference and to compare image quality and diagnostic confidence between the modalities.

## Materials and methods

### Study design and patient selection

We conducted a prospective single-center study in accordance with the Declaration of Helsinki in its current version. The institutional review board of our clinic approved the design of the trial (ethics proposal number 21–0763). Written informed consent was obtained from all included subjects.

We screened patients admitted to the emergency department, between May 10, 2021, and March 5, 2023, with clinically suspected fractures of the wrist and/or hand, or radiographically confirmed fractures, who received a CT scan as part of their routine clinical diagnostic work-up or for additional therapy planning, for eligibility. The exclusion criteria included contraindications for MRI (claustrophobia, non-MRI-compatible implants) and lack of consent to participate in the study. Eligible patients were enrolled and received an MRI scan of the wrist and/or hand within a maximum interval of 10 days from the CT scan. Additionally, we retrospectively searched for patients without a fracture in our picture archiving and communication system (PACS; Visage Client Version 7.1.18, Visage Imaging GmbH, Berlin, Germany) who received the study sequence (3D T1GRE) as part of an MRI due to other clinical issues (e.g. ulna impaction, osteoarthritis, tendinitis). These MRIs were re-examined for fractures by NH taking into account the clinical question and the standard MR sequences, including intermediate weighted turbo spin echo fat-saturated sequences.

### CT imaging

CT scans of the hand and/or wrist were performed either on a Revolution ACT or Optima CT660 scanner (both GE Medical Systems, Germany) using the following parameter settings: collimation, 0.625 mm; pixel spacing, 0.793 mm; pitch factor, 0.516; tube voltage, 120 kV; modulated tube current, 100–220 mA. Images were acquired in axial orientation and reformatted in sagittal, coronal and possibly additional (e.g. tilting along the longitudinal axis of the scaphoid) orientation using a bone-specific convolution kernel, slice thickness 1 mm. CT scans took 5 min including patient positioning and image reconstruction.

### MR imaging

MRI scans of the hand and/or wrist were performed on a 3-T MRI scanner (Magnetom Prisma, Siemens Healthineers, Erlangen, Germany) with a dedicated 16-channel wrist coil (Siemens Healthineers, Erlangen, Germany).

The protocol included the following sequences and took 21 min including patient positioning and multiplanar image reconstruction:

#### Study sequence (CT-like MRI)

- A T1-weighted three-dimensional gradient echo sequence with the following parameters: echo time, 3.54 ms; repetition time, 9.7 ms; flip angle, 8°; field of view, 90 × 90 mm^2^; matrix, 224 × 224; voxel size (acquisition = reconstruction), 0.4 × 0.4 × 0.4 mm^3^; acquisition duration, 4:24 min. The 3D sequence was acquired in coronal orientation and reformatted in coronal, axial and sagittal orientation with a slice thickness of 2.0 mm.

#### Clinical routine sequences

- A two-dimensional intermediate weighted turbo spin echo fat-saturated (IM fs) sequence in coronal orientation with the following parameters: echo time, 32 ms; repetition time, 3000 ms; flip angle, 150°; field of view, 107 × 120 mm^2^; matrix, 300 × 336; pixel dimension 0.36 × 0.36 mm^2^; slice thickness, 2.0 mm (0.2-mm gap).
- A two-dimensional T1-weighted turbo spin echo sequence in coronal orientation with the following parameters: echo time, 11 ms; repetition time, 500 ms; flip angle, 150°; field of view, 109 × 120 mm^2^; matrix, 348 × 384; pixel dimension, 0.31 × 0.31 mm^2^; slice thickness, 2.0 mm (0.2-mm gap).
- A two-dimensional IM fs sequence in axial orientation with the following parameters: echo time, 43 ms, repetition time, 4100 ms; flip angle, 180°; field of view, 72 × 105 mm^2^; matrix, 384 × 238; pixel dimension, 0.30 × 0.27 mm^2^; slice thickness, 2.2 mm (0.2-mm gap).
- A two-dimensional IM fs sequence in sagittal orientation with the following parameters: echo time, 38 ms, repetition time, 3350 ms; flip angle, 120°; field of view, 120 × 80 mm^2^; matrix, 256 × 346; pixel dimension, 0.35 × 0.31 mm^2^; slice thickness, 2.5 mm (0.2-mm gap).

For this study, only the CT-like MRI sequence was evaluated. The remaining sequences were evaluated as part of the clinical routine.

### Image analysis

For analysis, the T1GRE MR images were intensity-inverted to resemble CT images with a bright depiction of bony structures. These CT-like MR images as well as the conventional CT images were independently analyzed in random order by two radiologists (NH and JES, both radiologists with about 10 years of experience) blinded to clinical information and results from other modalities including radiographs using a picture archiving and communication system (PACS; Visage Client Version 7.1.18, Visage Imaging GmbH, Berlin, Germany). Prior to image analysis, the radiologists received instructions on fracture definition criteria using image examples. Fractures were defined as a visible break or interruption in the smooth cortical bone surface and/or disruption of the spongiosa pattern analogous to the signs of fracture known from CT. Bone marrow oedema, which appears as a homogeneous signal increase on CT-like MRI, without an accompanying fracture line was not considered sufficient for fracture detection.

CT images and CT-like MR images, respectively, were evaluated for the presence and location of wrist and/or hand fractures and graded according to the Arbeitsgemeinschaft Osteosynthese (AO German osteosynthesis working group) classification [17]. If more than one fracture was present, fractures were classified individually. The overall image quality of CT and CT-like MRI scans and the subjective diagnostic confidence were assessed by both raters on a 5-point Likert scale (1 = excellent, 2 = good, 3 = fair, 4 = below average, 5 = poor).

After completing the individual scan evaluation, both radiologists conducted a joint consensus reading to detect and classify fractures in the CT examinations. The results of this consensus CT reading served as the standard of reference for the statistical analysis of fracture detection and the AO classification.

**Fig. 1:**
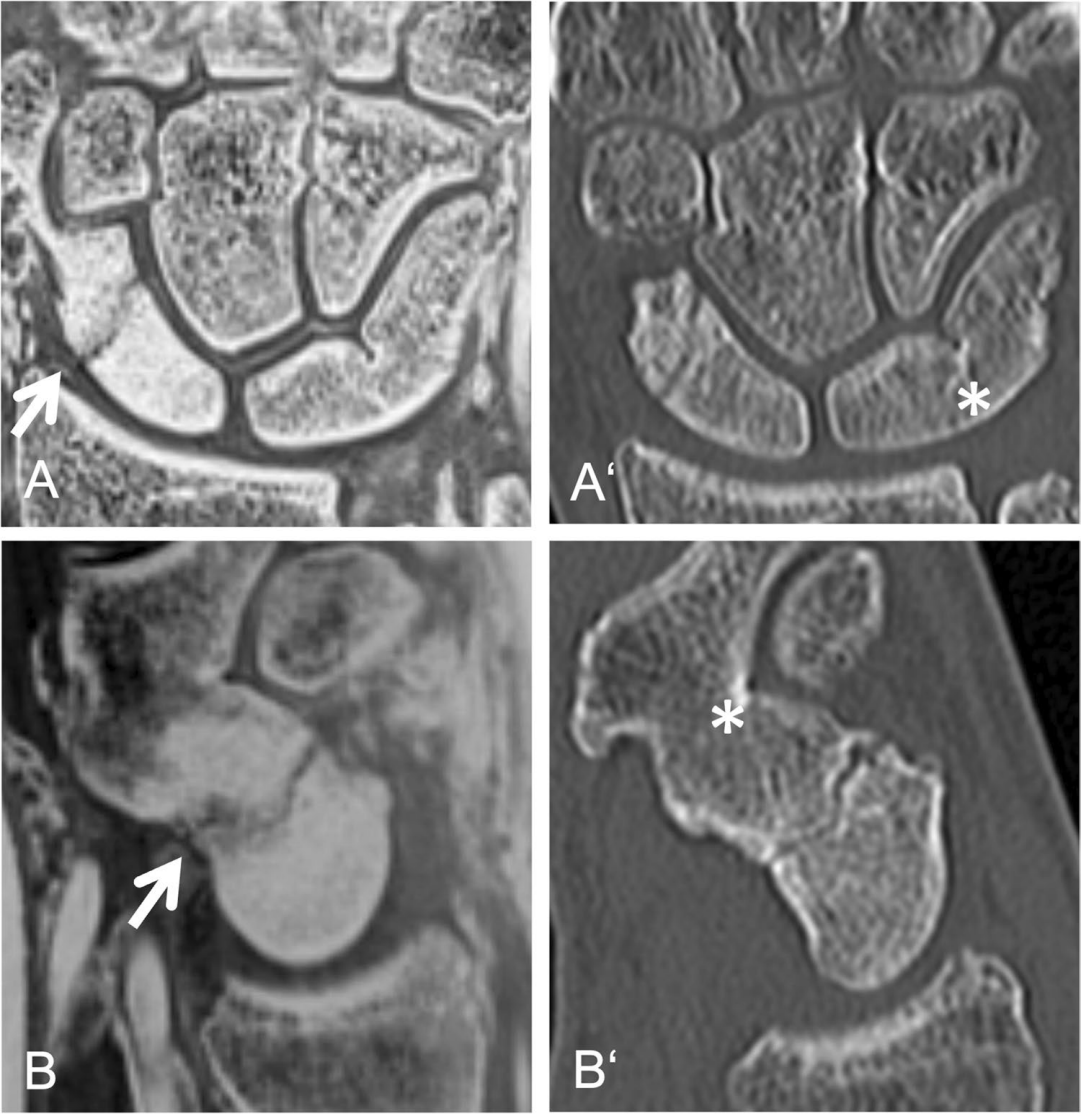
Twenty-three-year-old patient with an acute traumatic scaphoid fracture (arrow). Multiplanar reconstruction in coronal (A, A‘) and parasagittal plane (B, B‘) based on an inverted 3D T1GRE sequence (A, B) and a computed tomography (CT) (A‘, B‘) of a. Note, the bright homogenous oedema adjacent to the fracture line in the CT-like image, not visible on CT. In addition, the patient shows an osseous lunotriquetral and a scaphotrapezial coalition (asterisk (*)). 3D T1GRE, 3-dimensional T1-weighted gradient-echo; CT, computed tomography

**Fig. 2:**
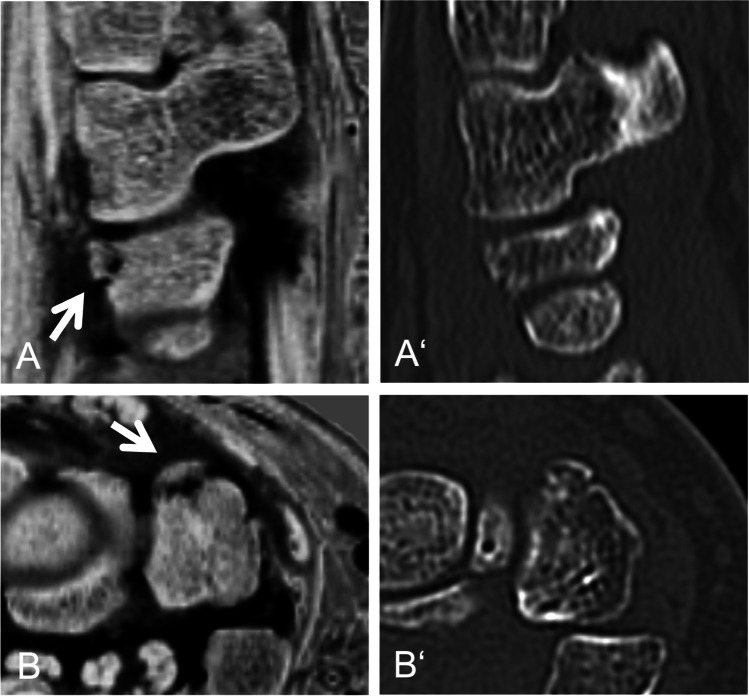
Forty-year-old patient with an acute traumatic avulsion fracture of the triquetrum (arrow). Multiplanar reconstruction in sagittal (A, A‘) and axial plane (B, B‘) based on an inverted 3D T1GRE sequence (A, B) and a CT (A‘, B‘). Note, the clearly depicted fracture line compared to the conventional CT in the axially reformated image (B‘)

**Fig. 3:**
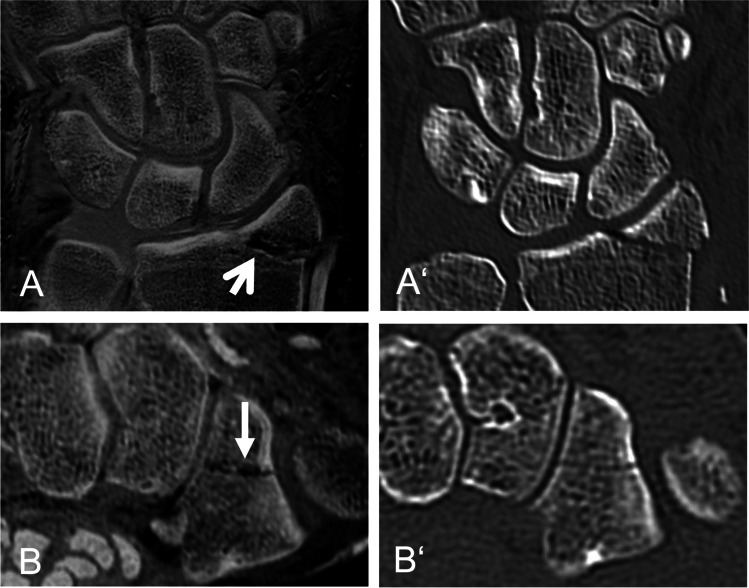
Twenty-three-year-old patient with an acute fracture of the styloid process of the radius (open arrow) and the trapezium (closed arrow). Multiplanar reconstruction in sagittal (A, A‘) and axial plane (B, B‘) based on an inverted 3D T1GRE sequence (A, B) and a CT (A‘, B‘)

**Fig. 4:**
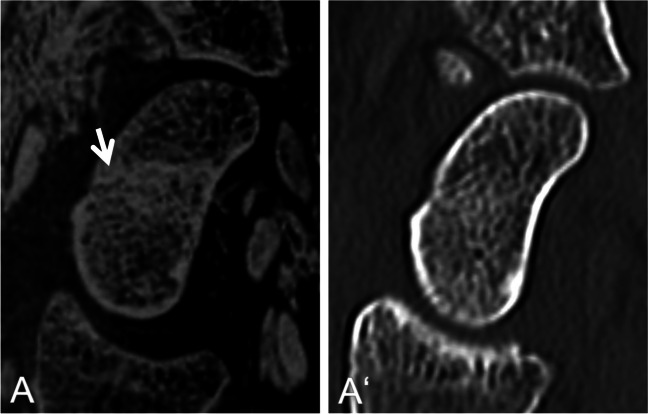
Twenty-six-year-old patient with a suspected fracture of the scaphoid (arrow). Multiplanar reconstruction in parasagittal plane (A, A‘) based on an inverted 3D T1GRE sequence (A) and a CT (A’). Fracture not clearly seen on CT. Time interval between CT and MRI 4 days. Note, the subtle fracture line with the adjacent oedema in A, rated as false positive

For image examples, please refer to the following figures: a scaphoid fracture (Fig. 1), a discrete triquetrum avulsion fracture (Fig. 2), a fracture of the distal radius and the trapezium (Fig. 3) and a scaphoid fracture suspected on CT-like MRI, which was not visible on CT images (Fig. 4).

### Statistical analysis

All data were analyzed using the SPSS (Statistical Package for the Social Science) Statistics® Software version 28.0 (IBM Corp., Armonk, USA).

Descriptive statistics were used to characterize the data. For comparison of image quality and overall diagnostic confidence, the Wilcoxon signed-rank test was used. Cohen’s Kappa was determined to assess the agreement of AO fracture classification between the modalities and between both readers. Sensitivity, specificity, positive predictive value, negative predictive value and accuracy were calculated to assess the performance of CT-like MRI compared to the reference standard (consensus reading CT) for fracture detection at the correct localization including cases with multiple fractures for both readers. The consensus CT reading served as a standard of reference for assessing the correct AO classification of fractures and fracture detection for both readers. The α level was at 0.05.

## Results

Seventy-three patients (46 females, age 38.84, standard deviation (SD) ± 14.27 years) were included in the study. Twenty-eight prospectively recruited patients (age 35.91 ± 14.2 years) had a total of 43 fractures (one patient had four fractures, two patients had three fractures, eight patients had two fractures). The predominantly retrospectively recruited control group without fracture (age 41.31 ± 14.00 years) consisted of 45 individuals, of which 16 had a CT. The mean time interval between CT and MRI was 5.37 ± 7.52 days in the fracture group and 16.25 ± 26.77 days in the control group (*p* = 0.61).

The frequencies of different fractures according to the AO classification are listed in Table 1.

**Table 1:** Frequency of different fractures in our cohort according to the AO classification. AO, Arbeitsgemeinschaft Osteosynthese

AO classification	Description	Number of fractures (*n*)
2R3A1	Avulsion fracture of the ulnar styloid process	1
2R3A2.1	Extra-articular simple fracture of the distal radius without malalignment	1
2R3B1.1	Partial intra-articular sagittal fracture of the distal radius through the scaphoid fossa	2
2R3B1.2	Partial intra-articular sagittal fracture of the distal radius through the lunate fossa	1
2R3B2.2	Partial intra-articular, multifragmentary dorsal edge fracture of the distal radius	2
2R3B2.3	Partial intra-articular dorsal edge fracture of the distal radius with displacement	1
2R3B3.2	Partial intra-articular, multifragmentary palmar edge fracture of the distal radius	2
71A	Avulsion fracture of the lunate bone	2
71B	Simple fracture of the lunate bone	1
72A	Avulsion fracture of the scaphoid bone	1
72B	Simple fracture of the scaphoid bone	9
73A	Avulsion fracture of the capitate bone	1
73B	Simple fracture of the capitate bone	1
74B	Simple fracture of the hamate bone	1
75A	Avulsion fracture of the trapezium bone	2
76.2A	Avulsion fracture of the triquetrum bone	7
76.2B	Simple fracture of the triquetrum bone	1
76.2C	Multifragmentary fracture of the triquetrum bone	2
77.2.1A	Extra-articular base fracture of the second metacarpal	1
77.2.1C	Completely intra-articular base fracture of the second metacarpal	1
77.3.1.A	Extra-articular base fracture of the third metacarpal	1
77.4.1.A	Extra-articular base fracture of the fourth metacarpal	1
78.1.3.1B	Partial intra-articular base fracture of the distal phalanx of the thumb	1

There was no significant difference regarding the overall image quality (*p* = 0.65) and diagnostic confidence (*p* = 1.00) between CT-like MRI and CT images (Table 2).

**Table 2:** Comparison of overall qualitative assessments of the CT-like MRI and CT

	CT-like MRI	CT	*p–* value^3^
Image quality^1,2^	1.19 ± 0.37	1.22 ± 0.42	0.65
Overall diagnostic confidence^1,2^	1.28 ± 0.53	1.28 ± 0.55	1.00

Data is given as mean ± standard deviation

^1^Measured on a 4-point Likert scale: 5 = very poor, 4 = poor, 3 = moderate 2 = good and 1 = very good

^2^Assessed with the Wilcoxon signed-rank test

There was no significant difference in the diagnostic confidence for CT-like MRI compared to CT for both raters (rater 1: CT-like MRI, mean ± SD, 1.27 ± 0.55 vs. CT, mean ± SD, 127 ± 0.52; *p* = 1.00; rater 2: CT-like MRI, mean ± SD, 1.10 ± 0.36 vs. CT, mean ± SD, 1.17 ± 0.42; *p* = 0.36). The same is applied for the assessment of image quality between CT-like MRI and CT for rater 1 (CT-like MRI, mean ± SD, 1.29 ± 0.59 vs. CT, mean ± SD, 1.14 ± 0.39; *p* = 0.07) and rater 2 (CT-like MRI, mean ± SD, 1.27 ± 0.64 vs. CT, mean ± SD, 1.42 ± 0.79; *p* = 0.23), respectively.

Agreement between CT-like MRI and conventional CT images regarding the AO fracture classification was excellent for both readers (reader 1: κ, 0.89; 95% confidence interval (CI), 0.80–0.97 and reader 2: κ, 0.89; CI, 0.80–0.97). Interrater agreement for AO fracture classification was excellent for CT-like MRI (κ, 0.82; 95% CI, 0.70–0.93) and CT images (κ, 0.85; 95% CI, 0.75–0.94).

All 43 fractures were correctly detected by both readers on CT.

The results of the four-square panel for both readers for the fracture detection of CT-like MRI are listed in Tables 3 and 4.

**Table 3:** Fracture detection of CT-like MRI for reader 1

Reader 1: CT-like MRI		
	**No fracture**	**Fracture**
No fracture	47 (true negative)	4 (false positive)
Fracture	0 (false negative)	43 (true positive)

Sensitivity 1.00, specificity 0.92, accuracy 0.96, positive predictive value 0.91, negative predictive value 1.00

**Table 4:** Fracture detection of CT-like MRI for reader 2

Reader 2: CT-like MRI		
	**No fracture**	**Fracture**
No fracture	48 (true negative)	3 (false positive)
Fracture	1 (false negative)	42 (true positive)

Sensitivity 0.98, specificity 0.94, accuracy 0.96, positive predictive value 0.93, negative predictive value 0.98

In the case of multiple fractures, all fractures were correctly localized by both readers. According to the consensus reading of the fracture AO classification, reader 1 accurately classified 36 out of 43 fractures in CT-like MRI, compared to 36 out of 43 in CT. Reader 2 accurately classified 40 out of 43 fractures in CT-like MRI vs. 42 out of 43 in CT.

## Discussion

Our analyses revealed that CT-like MRI based on a 3D T1GRE sequence is suitable for the detection and classification of wrist and hand fractures.

Reading CT-like MRI provided equal image quality and diagnostic confidence compared to conventional CT scans. The sensitivity, specificity and accuracy of CT-like MRI for detecting fractures were excellent (reader 1: 1.00, 0.92, 0.96; reader 2: 0.98, 0.94, 0.96). Furthermore, there was an excellent agreement for AO fracture classification between both modalities and between the readers.

The CT-like MRI sequence used in this study has previously been reported to provide excellent image quality and diagnostic confidence for fracture detection in the spine, skull, mandibula and shoulder [10–13, 15]. While radiography remains the primary means of fracture diagnosis due to its cost-effectiveness, low radiation dose and ubiquitous availability, CT offers advantages over radiography in detecting subtle fractures, visualizing fracture displacement and aiding preoperative planning [18].

CT-like MRI has been proven to reliably detect fractures and even discrete bony avulsions such as bony Bankart lesions of the glenoid after a traumatic shoulder dislocation, subtle fractures of the skull and the tibial eminence in children [11, 13, 16]. Osteoligamentous avulsions in the skeleton of the hand are even more common than in these regions, where apparently small bone avulsions can have major functional complications [19]. Ulas et al. reported an improved specificity of erosion detection in hand arthritis with bone-like MRI based on susceptibility-weighted imaging [20, 21]

However, to the best of the authors’ knowledge, there have been no studies on fracture detection with bone-like MRI of the hand and wrist so far. Our study cohort included a representative cross-section of wrist fractures seen in routine clinical practice, including scaphoid fractures, discrete avulsion fractures of the triquetrum, as well as intra- and extra-articular fractures of the distal radius. Hand and wrist fractures were reliably detected with CT-like MRI (sensitivity > 97%). CT-like MRI’s specificity has been negatively influenced by the false positive rate due to the fact that CT occult/subtle fractures, such as non-displaced scaphoid fractures, become more visible after a few days due to resorption processes at the fracture site (Fig. 4). To minimize this influence, we made efforts to keep the interval between the MRI and CT scans as short as possible (mean time interval between CT and MRI in the fracture group 5.37 ± 7.52 days). In contrast to CT, where bone marrow oedema is not always visible, pronounced oedema may appear bright on inverted images on CT-like MRI, confirming the suspicion of a fracture (Fig. 1) and increasing the diagnostic confidence.

The sensitivity of MRI for detecting wrist and hand fractures was already very high in our study, although only the CT-like sequence was used for diagnosis. In clinical use, the CT-like sequence would typically be integrated into a more comprehensive MRI trauma protocol, including an oedema-sensitive sequence, which has the potential to further improve the sensitivity of the examination [22].

In addition to fracture detection, assessment of fracture morphology and displacement is essential for treatment planning. Our results showed excellent agreement for AO fracture classification between CT-like MRI and CT. This is consistent with the results of Feuerriegel et al., which show accurate agreement between CT and CT-like MRI for the classification of traumatic mandibular fractures and measurement of dislocation [15].

The isotropical, CT-like MRI sequence offers the advantage of multiplanar reconstruction, like a CT scan. Parasagittal and paracoronal multiplanar reconstructions along the longitudinal axis of the scaphoid are particularly helpful in the assessment and treatment planning of scaphoid fractures [23, 24].

Usually, standard sequences are acquired together with CT-like 3D T1GRE sequences. These sequences allow the detection of further injury patterns, including traumatic bone marrow oedema (“bone bruises”), as well as ruptured ligaments (e.g. scapholunate ligament rupture and TFCC injuries) and ruptured tendons.

## Limitations

This study has several limitations. First, the acquisition of the 3D T1GRE CT-like sequence requires a 3 T MRI scanner with a state-of-the-art gradient system. On older scanners, examination duration might be prolonged due to increased minimum TR. Furthermore, the 3D T1GRE sequence is susceptible to metal artefacts. CT should therefore continue to be the preferred examination method for patients with metal implants. Patients with metal implants were not included in our study.

Particularly in the context of emergency care, MRI is usually less accessible than CT and associated with higher costs. Data on the imaging cost-effectiveness of hand and wrist fractures is very limited. According to a cost-effectiveness study on radiographically occult scaphoid fractures by Karl et al. from 2015, the mean price readjusted to 2014 values based on the Medical Consumer Price Index of a radiograph, CT and MRI was $54 ± $14, $374 ± $166 and $521 ± $120, respectively [25]. In summary, the study concludes that utilizing advanced imaging for suspected scaphoid fractures despite negative radiographs is cost-effective due to its affordability, reliable diagnostic precision and better projected health outcomes compared to empiric cast immobilization. Choosing between CT and MRI depends on institutional expenses, the willingness of the patient to undergo a radiation-dependent modality and the specific diagnostic capabilities available locally. Although radiography remains the first-line imaging method for the detection of hand and wrist fractures, CT-like MRI combined with a single coronal IM fs could be used instead of CT for fracture diagnosis in case of inconclusive radiographs and assessment of fracture morphology in the clinical setting. Reducing the number of sequences would indeed lower the MRI cost by saving time and might reduce the waiting period for MRI appointments.

In addition, the longer examination time compared to CT requires good patient compliance, and the MRI is contraindicated for individuals with certain implants, such as pacemakers. Therefore, the use of the proposed method may be limited to centers with the necessary resources and/or to patients who meet the above requirements.

3D T1GRE cannot distinguish between bone and ligaments, both appearing bright on grey inverted reformatted images. This may lead to false positive findings of avulsion fractures in the carpus due to the close proximity of tendons and bones. Crystal deposition associated with hydroxyapatite crystal deposition disease or calcium pyrophosphate dihydrate crystal deposition disease may also appear bright on intensity-inverted reformatted images, leading to false positive findings, but we had none of these cases in our cohort [26, 27].

## Conclusion

This study shows the effective diagnostic performance of CT-like MRI in the assessment of fractures in the wrist and hand compared to CT, the standard of reference, with comparable image quality and diagnostic confidence. The routine use of CT-like MRI in emergency diagnostics for suspected fractures of the wrist and hand has the potential to reduce the number of CT scans and the associated radiation exposure. Further research is needed to evaluate the potential impact on healthcare costs and MRI waiting times.

## Data Availability

The data that support the findings of this study are not openly available due to reasons of sensitivity of human data and are available from the corresponding author upon reasonable request.

## Abbreviations

CT: Computed tomography
MRI: Magnetic resonance imaging
3D T1GRE: 3D T1-weighted gradient echo sequence
IM fs: Intermediate weighted fat-saturated sequence
AO: Arbeitsgemeinschaft Osteosynthese
SD: Standard deviation
